# Megalocytivirus Induces Complicated Fish Immune Response at Multiple RNA Levels Involving mRNA, miRNA, and circRNA

**DOI:** 10.3390/ijms22063156

**Published:** 2021-03-19

**Authors:** Qian Wu, Xianhui Ning, Li Sun

**Affiliations:** 1 CAS and Shandong Province Key Laboratory of Experimental Marine Biology, CAS Center for Ocean Mega-Science, Institute of Oceanology, Chinese Academy of Sciences, 7 Nanhai Road, Qingdao 266071, China; wuqian@qdio.ac.cn (Q.W.); xhning@qdio.ac.cn (X.N.); 2 Laboratory for Marine Biology and Biotechnology, Qingdao National Laboratory for Marine Science and Technology, 1 Wenhai Road, Qingdao 266237, China; 3 College of Earth and Planetary Sciences, University of Chinese Academy of Sciences, 19 Yuquan Road, Beijing 100049, China

**Keywords:** *Paralichthys olivaceus*, megalocytivirus, miRNA, ceRNA, immune response

## Abstract

Megalocytivirus is an important viral pathogen to many farmed fishes, including Japanese flounder (*Paralichthys olivaceus*). In this study, we examined megalocytivirus-induced RNA responses in the spleen of flounder by high-throughput sequencing and integrative analysis of various RNA-seq data. A total of 1327 microRNAs (miRNAs), including 368 novel miRNAs, were identified, among which, 171 (named DEmiRs) exhibited significantly differential expressions during viral infection in a time-dependent manner. For these DEmiRs, 805 differentially expressed target mRNAs (DETmRs) were predicted, whose expressions not only significantly changed after megalocytivirus infection but were also negatively correlated with their paired DEmiRs. Integrative analysis of immune-related DETmRs and their target DEmiRs identified 12 hub DEmiRs, which, together with their corresponding DETmRs, formed an interaction network containing 84 pairs of DEmiR and DETmR. In addition to DETmRs, 19 DEmiRs were also found to regulate six key immune genes (mRNAs) differentially expressed during megalocytivirus infection, and together they formed a network consisting of 21 interactive miRNA-messenger RNA (mRNA) pairs. Further analysis identified 9434 circular RNAs (circRNAs), 169 of which (named DEcircRs) showed time-specific and significantly altered expressions during megalocytivirus infection. Integrated analysis of the DETmR-DEmiR and DEcircR-DEmiR interactions led to the identification of a group of competing endogenous RNAs (ceRNAs) constituted by interacting triplets of circRNA, miRNA, and mRNA involved in antiviral immunity. Together these results indicate that complicated regulatory networks of different types of non-coding RNAs and coding RNAs are involved in megalocytivirus infection.

## 1. Introduction

Megalocytivirus is an important viral pathogen to a wide range of aquaculture fish, including Japanese flounder (*Paralichthys olivaceus*), a valued marine fish. Megalocytivirus is a double-stranded DNA virus belonging to the family *Iridoviridae*. To date, several types of megalocytivirus have been identified, including infectious spleen and kidney necrosis virus (ISKNV), red seabream iridovirus (RSIV), rock bream iridovirus (RBIV), orange-spotted grouper iridovirus (OSGIV), and turbot reddish body iridovirus (TRBIV) [1,2,3,4,5]. Following infection of the host fish, megalocytivirus induces hemorrhage and the production of hypertrophied cells in various organs, particularly the lymphoid tissues [6,7]. In Japanese flounder, reports have shown that megalocytivirus infection elicits systematic changes in the expression of small non-coding RNAs and mRNAs in the spleen [8]. 

MicroRNAs (miRNAs) are a class of small endogenous RNAs ranging 21–24 nt in size and play an important role in mRNA translation. MicroRNAs execute post-transcriptional regulation of their target gene expression by binding to the 3′ untranslated region (3′-UTR) of the target mRNAs, which leads to blockage of mRNA translation [9]. Accumulating studies have indicated that miRNA-mediated regulation is involved in many biological processes, including inflammation, disease, and immune response to pathogen infection [9,10]. MicroRNAs have an important role in modulating the replication and pathogenesis of mammalian and fish viruses, such as dengue virus (DENV), West Nile virus (WNV), megalocytivirus, and red-spotted grouper nervous necrosis virus (RGNNV) [11,12,13]. MicroRNAs can also mediate anti-viral immune responses by targeting specific host immune genes, whereby activating or inhibiting the downstream signaling pathways [14,15]. 

Circular RNAs (circRNAs) are a novel type of endogenous noncoding RNAs that form covalently closed continuous loops without 3′ and 5′ ends [16,17]. Exon-derived circRNAs are generated by back-splicing, in which a 5′ splice donor attacks an upstream 3′ splice site, leading to a 3′–5′ phosphodiester bond that generates a circular RNA molecule [18]. Circular RNAs are ubiquitously expressed in eukaryotic cells and critical in many physiological and pathological conditions [19,20,21,22]. Circular RNAs can affect gene expression through diverse mechanisms, such as transcription and splicing regulation, microRNA (miRNA) sponges, mRNA traps, translational modulation, and post-translational modification [23]. In mammals, studies have provided emerging evidence that circRNAs play vital roles in various innate immune responses, including that associated with viral infection [24,25,26,27]. In fish, studies on the antiviral effect of circRNA is limited [28,29].

RNA sequencing (RNA-seq) is a recently developed method that uses deep-sequencing technologies for RNA profiling [30]. RNA-seq has been used widely in the study of pathogen associated immune response in fish [31,32]. In the present study, by employing the high-throughput sequencing technique and integrative analysis approaches, we depicted the expression profiles and interactive networks of flounder miRNAs and circRNAs at different stages of megalocytivirus infection. The differentially expressed miRNAs (DEmiRs) and circRNAs (DEcircRs) were identified and analyzed with the methods of Gene Ontology (GO) and Kyoto Encyclopedia of Genes and Genomes (KEGG) enrichment. On the basis of the previously reported mRNA transcriptome data [33], the differentially expressed target mRNAs of the miRNAs (DEmRs) were also identified. Interactive networks of immune-related DEmiRs and their differentially expressed target mRNAs (DETmRs), as well as interactive circRNAs-miRNAs-mRNAs, were constructed. 

## 2. Results

### 2.1. High-Throughput Sequencing and Quality Assessment 

**Figure 1:**
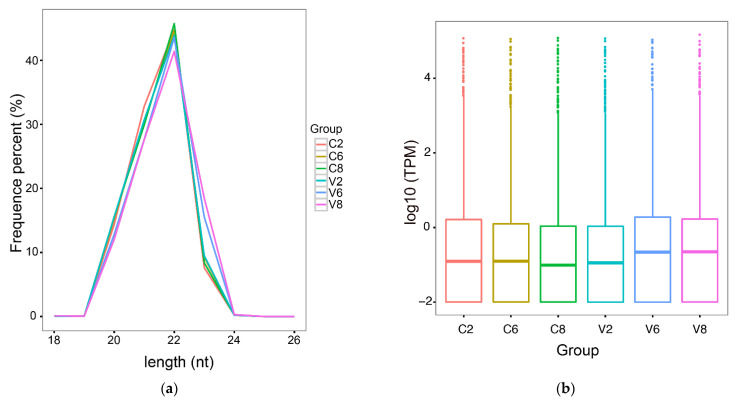
MicroRNA (miRNA) sequencing data analysis in the spleen of flounder. (**a**) The length distribution of the miRNAs in each group. “C2”, “C6”, and “C8” indicate control fish groups at 2, 6, or 8 days post infection (dpi), respectively; “V2”, “V6”, and “V8” indicate megalocytivirus-infected fish groups at 2, 6, or 8 dpi, respectively; (**b**) Boxplot profiling the expression levels of the miRNAs in each group at 2, 6, and 8 dpi. TPM, transcripts per million.

In the micro-transcriptome analysis, 18 libraries were constructed with RNAs from three groups (3 fish/group) of megalocytivirus-infected fish at 2, 6, and 8 dpi (named V2d, V6d, and V8d, respectively) and three control groups (3 fish/group) of uninfected fish at the corresponding time points (named C2d, C6d, and C8d, respectively). A mean number of 11,855,921 filtered clean tags were obtained from each library (Table 1). On the basis of the flounder genome, a total of 1327 miRNAs were identified, including 959 known miRNAs and 368 novel miRNAs (Table 1). The lengths of the miRNAs in all six groups were distributed in the range 20–23 nt, with a maximum of 22 nt, indicating a high degree of consistency between groups (Figure 1a). The expression levels of the miRNAs in the control and virus-infected groups are shown in a boxplot (Figure 1b). 

### 2.2. Differentially Expressed miRNAs (DEmiRs) Induced by Megalocytivirus 

**Figure 2:**
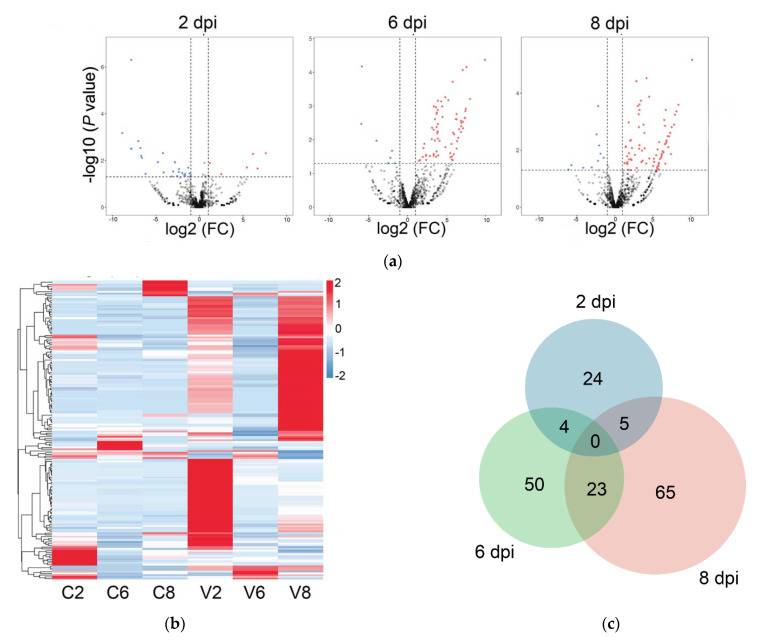
Analysis of differentially expressed miRNAs (DEmiRs) at different time points of infection. (**a**) Volcano plots of DEmiRs, at 2, 6, or 8 days post infection (dpi). The red and blue points represent up- and downregulated miRNAs, respectively; gray points represent miRNAs without significant change. FC, fold change; (**b**) The heatmap of DEmiR expression profiles in different groups at different time points. For convenience, “C2”, “C6”, and “C8” indicate the control groups at 2, 6, and 8 dpi, respectively; “V2”, “V6”, and “V8” indicate megalocytivirus-infected groups at 2, 6, and 8 dpi, respectively. (**c**) Venn diagram showing the overlap of DEmiRs at 2 dpi (blue), 6 dpi (green), and 8 dpi (pink).

**Figure 3:**
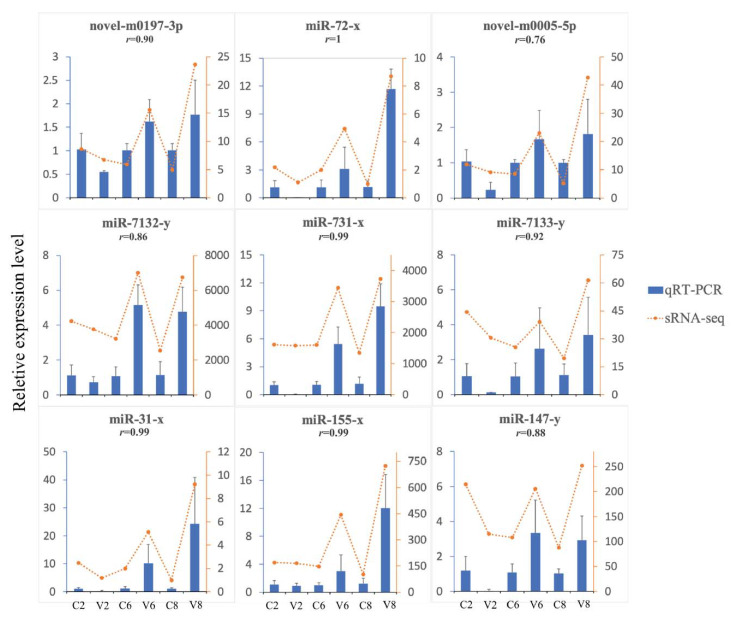
Validation of the small RNA-seq data by quantitative real-time PCR (qRT-PCR). The relative expression profiles of 9 DEmiRs, at 2, 6, or 8 days post infection, detected by qRT-PCR (bars) and compared with the results of RNA-seq (dotted lines). Error bars represent standard deviations. For each DEmiR, the correlation coefficient (r) between the results of qRT-PCR and sRNA-seq is shown.

Compared with the control fish, 171 miRNAs exhibiting significantly differential expressions during viral infection were detected and named DEmiRs. Of these DEmiRs, 159 were upregulated and 44 were downregulated. The numbers of DEmiRs at 2, 6, and 8 dpi were 33, 77, and 93, respectively (Table 2). The Volcano plots of the DEmiRs at the three time points are shown in Figure 2a. There were more downregulated DEmiRs than upregulated DEmiRs at 2 dpi, while the majority of DEmiRs at 6 dpi and 8 dpi were upregulated. The time-dependent expression profiles and the numbers of DEmiRs are shown in Figure 2b,c, respectively. In Figure 2c, 24, 50, and 65 DEmiRs were identified exclusively at 2, 6, and 8 dpi, respectively; four DEmiRs were identified at both 2 and 6 dpi; 23 DEmiRs were identified at both 6 and 8 dpi; five DEmiRs were identified at both 2 and 8 dpi; no DEmiRs were identified at all three time points. To verify the DEmiRs obtained by small RNA deep sequencing (sRNA-seq), the expressions of nine DEmiRs were tested by qRT-PCR. The results of qRT-PCR were consistent with that of sRNA-seq at 2, 6, and 8 dpi, with correlation coefficients ranging from 0.76 to 1.00 (Figure 3).

### 2.3. Identification and Analysis of the Targets of DEmiRs

A total of 27,796 putative target mRNAs were predicted for the 171 DEmiRs. These genes were further submitted to integrated analysis, by which the expressions of these genes were compared with that of their paired DEmiRs. As a result, among the 27,796 putative target mRNAs, 805 differentially expressed target mRNAs (DETmRs) were identified, whose expression levels not only significantly changed after megalocytivirus infection but also were negatively correlated with that of their paired DEmiRs. In addition to targeting the host mRNAs, 148 DEmiRs were predicted to target the mRNAs of megalocytivirus as well. 

**Figure 4:**
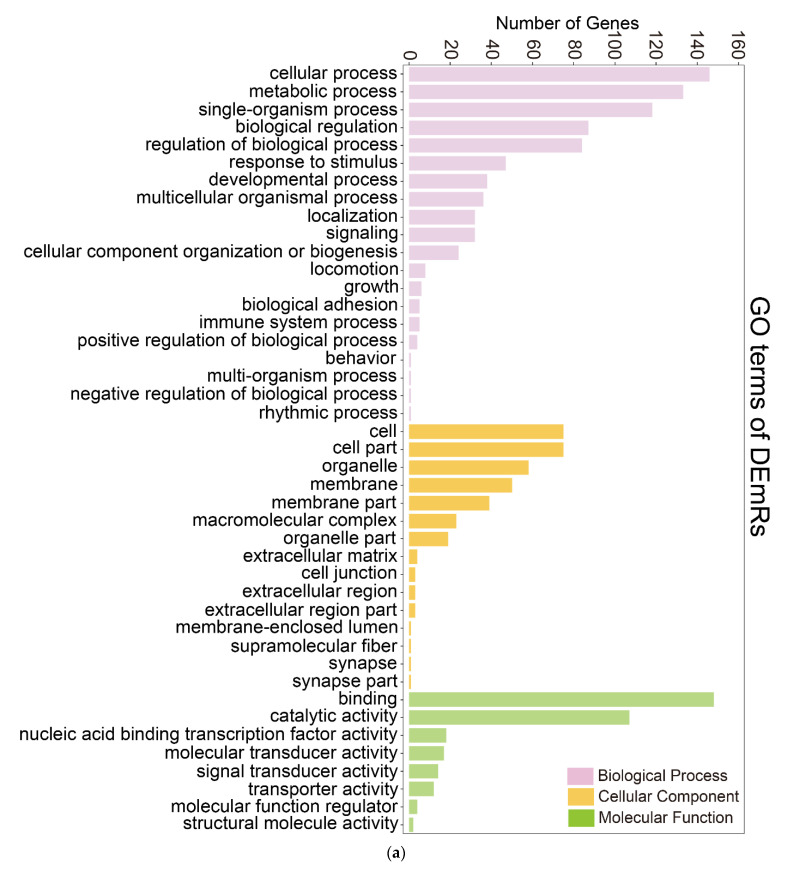

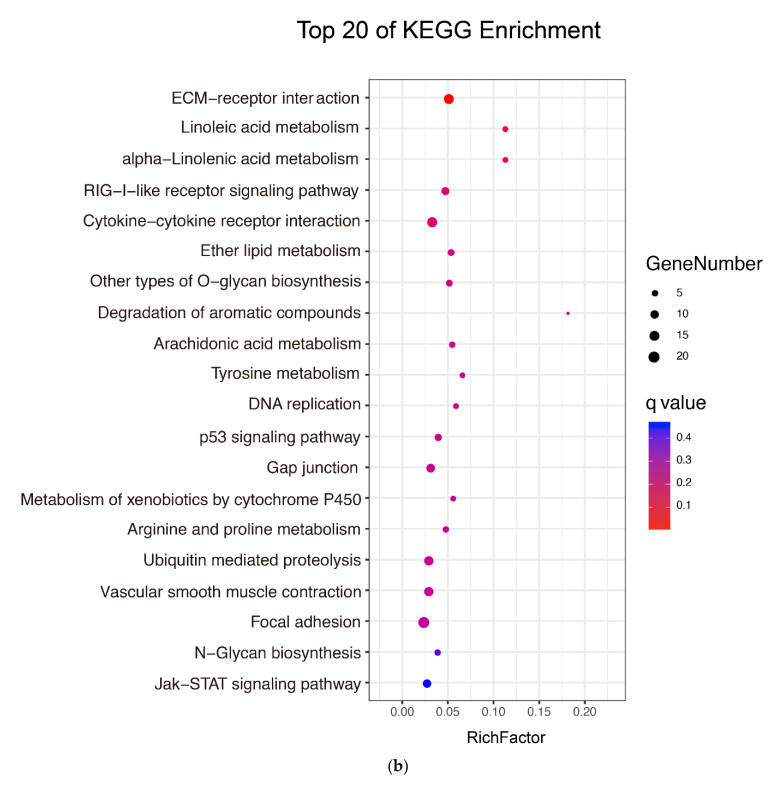
Functional enrichment of the differentially expressed target genes of DEmiRs (DETmRs). (**a**) DETmRs enriched in Gene Ontology (GO) terms; (**b**) The top 20 enriched Kyoto Encyclopedia of Genes and Genomes (KEGG) pathways of the DETmRs.

GO functional enrichment analysis categorized the DETmRs into three functional groups, i.e., biological process, cellular component, and molecular function (Figure 4a). In biological process, the DETmRs were enriched in “cellular process”, “metabolic process”, “single-organism process”, “biological regulation”, “regulation of biological process”, and other processes including “response to stimulus” and “immune system process”. In the cellular component category, the DETmRs were mainly classified into the groups associated with cell, organelle, and membrane. In the molecular function category, the DETmRs belonged mainly to binding and catalytic activity process. KEGG analysis showed that the top 20 enriched KEGG pathways included five immune-related pathways, i.e., RIG-I-like receptor signaling pathway, cytokine–cytokine receptor interaction, ubiquitin-mediated proteolysis, p53 signaling pathway, and JAK-STAT signaling pathway (Figure 4b).

### 2.4. The Interactive Network of Immune-Related DEmiRs-DETmRs

**Figure 5:**
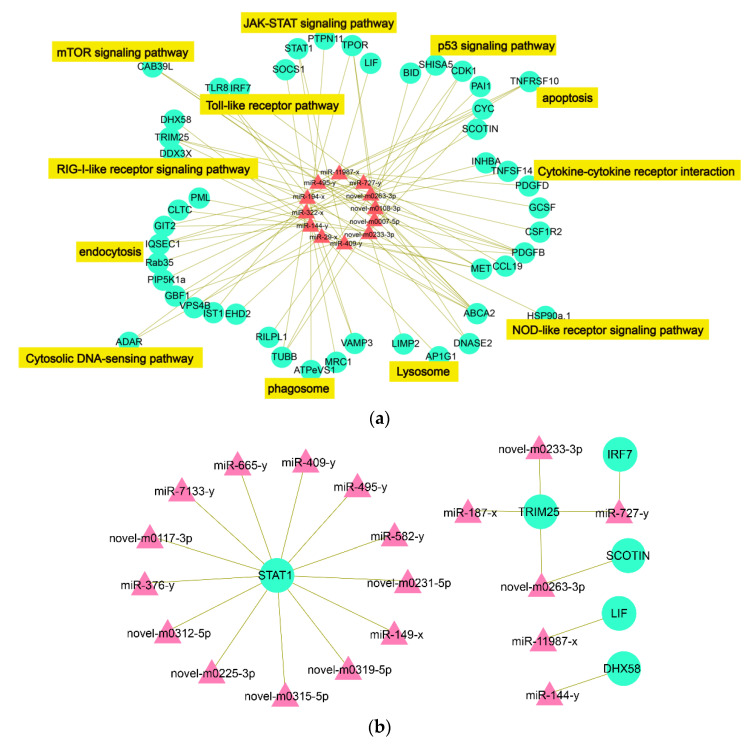
Immune-related miRNA-mRNA interaction networks. (**a**) The immune-related miRNA-mRNA network formed by interactive DEmiRs and DETmRs. The red triangle nodes indicate DEmiRs and the green round nodes indicate DETmRs. The pathways to which the DETmRs belong are highlighted in yellow; (**b**) The mRNA-miRNA networks formed by the key immune-related differential expressed genes (DEGs), identified in a previous mRNA transcriptome study, and their paired DEmiRs. The green round nodes indicate the key immune DEGs and the pink triangle nodes indicate the DEmiRs that target the hub genes of (**a**).

Forty-seven immune-related DETmRs were identified in the KEGG pathways and subjected to integrative analysis with DEmiRs, resulting in the identification of 12 DEmiRs, each of which interacted with more than five DETmRs (i.e., degree > 5). These DEmiRs were defined as hub DEmiRs. As shown in Table 3, the 12 hub DEmiRs exhibited differential expressions mostly at 8 dpi and interacted with 5 to 11 DETmRs. The hub DEmiRs and their paired immune-related DETmRs formed a DEmiR-DETmR interaction network consisting of 84 interacting DEmiR-DETmR pairs (Figure 5a). The DETmRs in the network were involved in various immune pathways (Figure 5a).

In a previous transcriptome analysis of the same samples used in this study, we identified 16 key immune-related and differentially expressed genes (DEGs) significantly induced by megalocytivirus [33]. In the present study, we found that six of these key immune-related DEGs, i.e., STAT1 (signal transducer and activator of transcription 1), TRIM25 (an E3 ubiquitin/ISG15 ligase), IRF7 (interferon regulatory factor 7), DHX58, SCOTIN, and LIF (leukemia inhibitory factor), were targeted by 19 DEmiRs. As shown in Figure 5b, the interaction network formed by the 19 DEmiRs and the six DEGs contained 21 interactive miRNA-mRNA pairs, and STAT1 is the DEG targeted by the largest number of DEmiRs (degree = 13). 

### 2.5. Overview of the circRNA Sequencing Data

**Figure 6:**
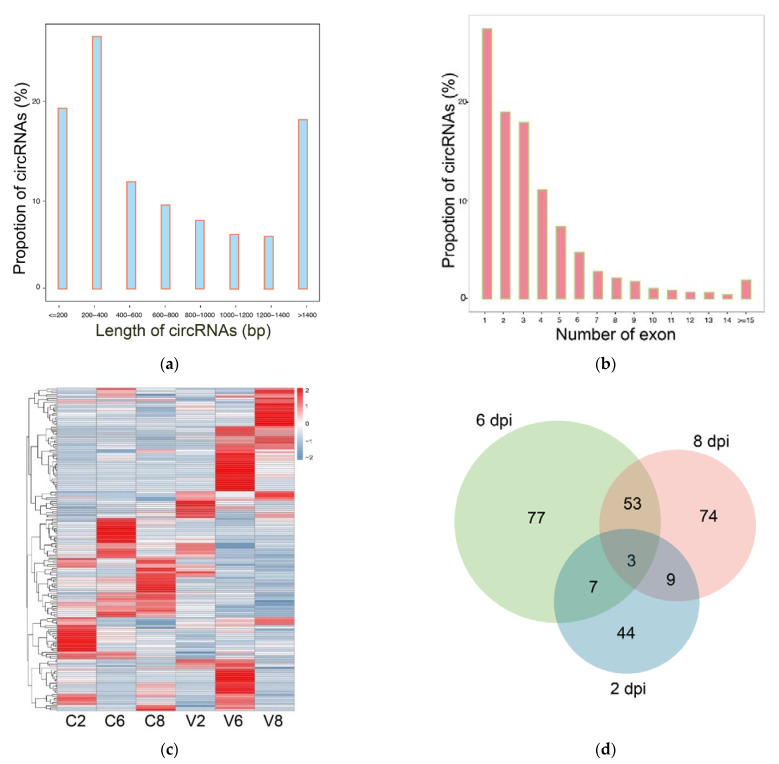
Analysis of circular RNA (circRNA) sequencing data. (**a**) The length distribution of circRNAs; (**b**) The exon number of circRNAs; (**c**) The heatmap of DEcircR expression profiles in different groups at different time points. For convenience, “C2”, “C6”, and “C8” indicate the control groups at 2, 6, and 8 dpi, respectively; “V2”, “V6”, and “V8” indicate megalocytivirus-infected groups at 2, 6, and 8 dpi, respectively; (**d**) Venn diagram showing the overlap of DEcircRs at 2 dpi (blue), 6 dpi (green), and 8 dpi (pink).

As abovementioned, the same megalocytivirus-infected and uninfected samples used in this study had been subjected to transcriptome analysis in a previous study [33]. In the current study, these transcriptome data were explored for circRNA information. After a series of selection, 9434 circRNAs were identified. The length of the circRNAs ranged from 53 to 96,180 bp, mostly within 200–400 bp (Figure 6a). The number of exons in the circRNA transcripts mainly ranged from 1 to 6 (Figure 6b). Expression analysis showed that 169 circRNAs exhibited significantly different expressions after megalocytivirus infection and were named DEcircRs. The expressions of the DEcircRs were time dependent, with 44, 77, and 74 DEcircRs occurring at 2, 6, and 8 dpi, respectively (Table 4). The expression profiles of all DEcircRs are shown in a heat map in Figure 6c. Venn diagram showed that seven DEcircRs occurred at 2 and 6 dpi, 53 DEcircRs occurred at 6 and 8 dpi, nine DEcircRs occurred at 2 and 8 dpi, and only three DEcircRs (circ_007062, circ_009376, and circ_008173) occurred at all three time points (Figure 6d).

### 2.6. Competing Endogenous RNAs (ceRNAs) 

**Figure 7:**
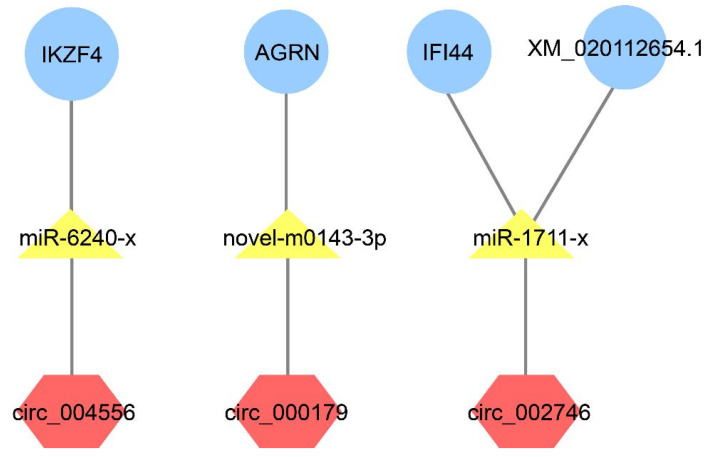
The predicted differentially expressed circRNA/miRNA/mRNA triplets. The yellow triangle nodes indicate the differentially expressed miRNAs (DEmiRs); the red hexagon nodes indicate the differentially expressed circRNAs (DEcircRs) that paired to the DEmiRs; the blue round nodes indicate the differentially expressed mRNAs (DEmRs) that paired to the DEmiRs.

It is known that circRNAs can act as ceRNAs to compete with mRNAs for miRNA interaction, whereby regulating the expression of corresponding genes [23]. Three softwares (Mireap, miRanda, and TargetScan) were used to predict the target relationship between DEcircRs and DEmiRs. The negatively and significantly correlated DEcircRs and DEmiRs were identified as regulatory DEmiR-DEcircR pairs. Seventeen DEmiR-DEcircR pairs were found, consisting of 14 DEcircRs and 14 DEmiRs. The integrated analysis of the interactions of DETmRs-DEmiRs and DEcircRs-DEmiRs resulted in the identification of four interacting triplets of circRNA, miRNA, and mRNA formed by three DEcircRs, three DEmiRs, and four DETmRs (Figure 7). Of the four DETmRs, IKZF4 (zinc finger protein Eos) plays an essential role in CD4+ regulatory T cells (Tregs) programming and affects multiple aspects of Treg suppressor function [34,35], AGRN (agrin) is involved in cervical tumourigenesis and sepsis-induced neuromuscular dysfunction [36,37], IFI44 (interferon-induced protein 44) can restrict viral activity and replication [38,39], and XM_020112654.1 is a uncharacterized protein with unknown function.

## 3. Discussion

In this study, deep Illumina sequencing and integrative analysis were performed to examine the expression of miRNAs and circRNAs in Japanese flounder infected by megalocytivirus. We found that megalocytivirus affected the expression of 171 miRNAs, which were predicted to target 805 DETmRs. The immune-related DETmRs were used for the construction of differentially expressed miRNA-mRNA interaction network, which contained 12 hub DEmiRs (degree > 5) and 47 DETmRs. A total of 169 DEcircRs were identified, which were significantly induced by megalocytivirus. Furthermore, based on integrative analysis, the differentially expressed ceRNAs regulatory units consisting of circRNA/miRNA/mRNA triplets were identified. The important DEmiRs, DETmRs, and DEcircRs involved in immune defense are discussed below.

### 3.1. Endocytosis and Lysosome

As shown in Figure 4a, the number of immune DETmRs enriched in the category of endocytosis is the largest. Endocytosis is used by various viruses to enter host cells [40]. For example, caveola-dependent endocytosis was used for ISKNV and Singapore grouper iridovirus (SGIV) to enter fish cells [41,42]. In our study, three hub DEmiRs, i.e., novel-m0233-3p, novel-m0236-3p, and miR-322-x, were found to target clathrin heavy chain (CLTC), Ras-related protein Rab35 (Rab35), and phosphatidylinositol 4-phosphate 5-kinase type-1 alpha (PIP5K1α), respectively. CLTC is an essential molecule in the process of clathrin-mediated endocytosis and required for viral invasion into host cells [43,44,45]. Rab35 is a plasma membrane-localized protein and controls a fast endocytic recycling pathway as well as autophagy [46,47]. PIP5K1α can influence the internalization and infection of various viruses, such as influenza A virus, foot-and-mouth disease virus, and vesicular stomatitis virus [48,49]. Similar to Rab35, PIP5K1α was also reported to participate in the process of autophagy [50]. These results indicate that, through their DETmRs, the hub DEmiRs likely modulate the endocytosis process to influence the internalization and intracellular trafficking of megalocytivirus in flounder.

Lysosomes are the terminal compartments in the endocytic pathway, where the pathogen internalized by endocytosis is eliminated [51,52]. In this study, we found that one hub DEmiR, i.e., miR-29-x, was significantly upregulated at 6 and 8 dpi. MiRNAs related to miRNA-29 are associated with resistance to HIV-1, hepatitis B virus, and hepatitis C virus infection [53,54,55]. In our study, miR-29-x was predicted to target the DETmR AP-1 complex subunit gamma-1 (AP1G1), which in mammals is known to affect HIV-1 infection by influencing the process of HIV-mediated CD4 internalization and targeting to lysosomes [56]. Another hub DEmiR, novel-m0233-3p, was predicted to target LIMP2 (lysosome membrane protein 2), which is located primarily in lysosomes and late endosomes and may participate in reorganizing the endosomal/lysosomal compartments [57]. A recent study showed that LIMP2 of turbot is implicated in the immune response to bacterial infection with unknown mechanism [58]. Another lysosome-associated membrane protein, ATP-binding cassette sub-family A member 2 (ABCA2), was predicted to be targeted by seven hub DEmiRs, including miR-194-x, which was shown to be associated with antiviral immune activity [59,60,61,62,63]. These results indicate that lysosomes probably play a critical role in megalocytivirus infection.

### 3.2. Toll- and RIG-I-Like Receptor Signaling Pathways 

Toll-like receptors (TLRs) and RIG-I-like receptors (RLRs) are classical pattern recognition receptors (PRRs) involved in viral interaction. TLRs and RLRs can recognize viral RNA and induce the expression of type I interferons (IFNs) and other inflammatory cytokines to limit viral replication in mammals and fish [64,65,66,67]. In our study, the RLR DHX58 was found to be targeted by the DEmiR miR-144-y, which was significantly downregulated during megalocytivirus infection. MiR-144-y is known to reduce antiviral response by attenuating the TRAF6-IRF7 pathway in influenza virus-infected mice [68]. The decreased expression of miR-144-y observed in our study implies elevated activity of RLRs, which may facilitate viral removal by enhancing IRF7 expression. Two DETmRs, i.e., TLR8 and DDX3X (ATP-dependent RNA helicase), were targeted by two and four hub DEmiRs, respectively. TLR8 has been shown to be associated with antiviral immunity in Zika virus-infected patients [69,70]. DDX3X and its regulatory miRNA, miR-322-x, were essential to promoting apoptosis in mouse spermatocyte GC-2 cells [71]. DDX3X could also regulate antiviral response by stimulating the production of IFN-I and supplementing the function of RIG-I and MDA-5 in the early phase of virus infection [72]. Together these results suggest that, in flounder, TLRs and RLRs are likely involved in the recognition and clearance of megalocytivirus.

### 3.3. Cytokine–Cytokine Receptor Interaction 

Cytokines released by innate immune cells are the first line of defense against infectious pathogens by promoting the movement of monocytes and activating immune responses [73,74]. In fish, cytokines play an important role in anti-pathogen immunity by regulating cell mobilization and promoting tissue remodeling [75,76,77]. In our study, the hub DEmiR miR-11987-x was predicted to target two DETmRs, CCL19 (C-C motif chemokine 19) and GCSF (granulocyte colony-stimulating factor). CCL19 is a critical regulator of T cell activation, immune tolerance, and inflammatory responses, and can influence the outcome of HIV infection [78,79]. In a mouse model of RSV (respiratory syncytial virus) infection, GCSF was found to mediate antiviral activity by regulating neutrophils recruitment and activation [79]. In flounder, overexpression of GCSF stimulated host defense against pathogenic bacteria [80]. These results suggest a role for CCL19 and GCSF in anti-megalocytivirus infection in flounder. Another cytokine receptor targeted by hub DEmiR in our study is the DETmR tumor necrosis factor ligand superfamily member 14 (TNFSF14). TNFSF14 is known to restrict herpes simplex virus (HSV) entry into host cells by competing with the HSV envelope glycoprotein D for binding to the vital entry mediator [81]. It is possible that TNFSF14 contributes to the immune defense of flounder against megalocytivirus infection.

### 3.4. JAK-STAT Signaling Pathway

The JAK-STAT pathway is located downstream of numerous cytokine receptors and transfers cytokine signaling [82]. Studies have shown that some viruses escape host immunity by interfering with cytokine-mediated JAK-STAT signaling [83,84]. In our study, several DETmRs, including signal transducer and activator of transcription 1 (STAT1), suppressor of cytokine signaling 1 (SOCS1), tyrosine-protein phosphatase non-receptor type 11 (PTPN11, also called SHP2), and thrombopoietin receptor (TPOR), were enriched in the JAK-STAT pathway. STAT1, which was identified previously as a key immune-related gene regulated by megalocytivirus [33], was targeted by the hub DEmiR miR-409-y, which was strongly upregulated by megalocytivirus. In mice, miR-409-y is known to modulate the expression of SOCS3 and STAT3, as well as to induce the production of inflammatory cytokines [85]. The upregulated expression of miR-409-y observed in our study suggests a downregulated inflammatory response that may be advantageous to viral infection. 

SOCS1 is an inhibitor of JAK-STAT signaling and the interferon gamma (IFNγ) pathway [86]. Research has shown that transmissible gastroenteritis virus (TGEV) evaded from the type I interferon response through the miR-30a-5p/SOCS1 axis, and TGEV infection resulted in decreased miR-30a-5p level and elevated SOCS1 [87]. In our study, SOCS1 was a target of the hub DEmiR miR-144-y, which was significantly inhibited by megalocytivirus, suggesting that the virus might regulate SOCS1 through miR-144-y as an immune evasion mechanism. In addition to SOCS1, miR-144-y also targeted PTPN11, a negative regulator of JAK-STAT signaling that also participates in apoptotic activation [88,89,90]. Another DETmR implicated in the JAK-STAT signaling pathway was TPOR, which was targeted by three hub DEmiRs, suggesting a potentially important role of TPOR in megalocytivirus infection. 

## 4. Materials and Methods

### 4.1. Sample Collection 

In a previous study, we examined the mRNA transcription profiles of flounder infected by megalocytivirus at different times by sequencing 18 libraries of spleen samples from megalocytivirus-infected and uninfected fish, at 2, 6, or 8 days post infection (dpi) [33]. Briefly, clinically healthy Japanese flounder (~250 g) were divided randomly into two groups (36 fishes/group) and injected intraperitoneally with megalocytivirus RBIV-C1 or PBS (control). The fish were sampled at 2, 6, and 8 dpi (3 fish/time point), and spleen from each of the fish was taken and used as an individual sample for RNA sequencing described below. 

### 4.2. Small RNA Sequencing 

For small RNA sequencing, total RNA was extracted by Trizol reagent (Invitrogen, Carlsbad, CA, USA) from the above 18 samples (9 samples of infected fish and 9 samples of control fish). The quality of the isolated RNAs was evaluated using NanoDrop Spectrometer ND-2000 (Thermo Fisher Scientific, Waltham, MA, USA) and Agilent 2100 bioanalyzer (Agilent Technologies, Palo Alto, CA, USA). The RIN values of the 18 samples ranged from 8.6 to 10. The RNA molecules in a size range of 18–30 nt were enriched by polyacrylamide gel electrophoresis (PAGE). Then, the 3′ adapters and 5′ adapters were ligated. The ligation products were reverse transcribed and amplified by PCR. There are 12 Index Primers for producing barcoded libraries in the NEBNext^®^ Multiplex Small RNA Library Prep Set for Illumina^®^ (Set 1) kit (NEB #E7300L, NEB, Ipswich, MA, USA). For each PCR reaction, only one of the 12 Index Primers was used. The 140–160 bp PCR products were enriched to generate a cDNA library, which was sequenced using Illumina HiSeqTM 2500 Gene Denovo Biotechnology Co. (Guangzhou, China). 

### 4.3. Data Processing 

The raw reads were filtered to get clean tags by removing reads that contained more than one low quality (Q value ≤ 20) base, unknown nucleotides, without 3′ adapters, containing 5′ adapters, containing 3′ and 5′ adapters but no small RNA fragment between them, containing ployA in small RNA fragment, and shorter than 18 nt. All the clean tags were aligned with the small RNAs in GeneBank database (Release 209.0) and Rfam database (11.0) (http://rfam.xfam.org (accessed on 24 December 2018)) to identify and remove rRNA, scRNA, snoRNA, snRNA, and tRNA. The clean tags were also aligned with the reference genome of flounder (GenBank project accession PRJNA369269) via Bowtie (v1.1.2) [91]. The tags mapped to exons or introns might be fragments from mRNA degradation, and therefore were removed. The tags mapped to repeat sequences were also removed. 

### 4.4. MiRNA Identification and Differential Expression Analysis

The remained clean tags were searched against miRBase database (Release 21) to identify known miRNAs in Japanese flounder. Meanwhile, novel miRNAs were predicted according to their genome positions and hairpin structures using the software MIREAP (v0.2) [92]. Total miRNA expression level was calculated and normalized to transcripts per million (TPM). The edgeR package (v3.12.1) (http://www.r-project.org/ (accessed on 11 January 2019)) was used to identify differentially expressed miRNAs (DEmiRs), with a fold change (FC) > 2 (log2|FC| > 1) and *p* < 0.05, between the infected and uninfected groups at 2, 6, and 8 dpi. 

### 4.5. CircRNA Identification and Differential Expression Analysis

For circRNA identification, high quality clean reads were obtained by further filtering the raw reads according to the following rules: removing reads containing adapters, removing reads containing more than 10% of unknown nucleotides (N), and removing low quality reads containing more than 50% of low quality (Q value ≤ 20) bases. Next, Bowtie2 was used for mapping reads to ribosome RNA (rRNA) database, and the rRNA mapped reads were removed. The remaining reads of each sample were mapped to the reference genome of Japanese flounder by TopHat2 (version 2.0.3.12). After aligning with the reference genome, the reads that were continuously mapped to the genomes were discarded, and the unmapped reads were collected for circRNA identification; 20 mers from both ends of the unmapped reads were extracted and aligned to the reference genome to find unique anchor positions within splice site. Anchor reads that aligned in the reversed orientation (head to tail) indicated circRNA splicing and were subjected to find_circ [19] to identify circRNAs. Then, the anchor alignments were extended such that the completely read aligns and the breakpoints were flanked by GU/AG splice sites. A candidate circRNA was identified if it was supported by at least two unique back spliced reads in at least one sample. The identified circRNAs were subjected to statistical analysis of type and length distribution. To identify differentially expressed circRNAs across groups, the edgeR package (http://www.rproject.org/ (accessed on 11 January 2019)) was used. The circRNAs with a fold change ≥ 2 and a *p* value < 0.05 between groups were defined as significantly and differentially expressed circRNAs (DEcircRs). 

### 4.6. Identification and Functional Enrichment Analysis of the DETmRs

The predicted target genes of DEmiRs were obtained based on the intersecting results of the analyses using three softwares, i.e., RNAhybrid (v2.1.2) + svm_light (v6.01), Miranda (v3.3a), and TargetScan (v7.0), with default parameters. These predicted target genes were subjected to integrated analysis of miRNA-mRNA expressions using the miRNA expression data in this study and the mRNA expression data reported previously for the same samples [33]. The predicted target genes that were differentially expressed and negatively correlated in expression with their paired DEmiRs were considered to be differential expressed target genes (DETmRs). The DETmRs were functionally annotated using the GO database (http://geneontology.org (accessed on 28 December 2018)) and KEGG database (http://www.genome.jp/kegg/ (accessed on 28 December 2018)). Through hypergeometric test, GO terms and KEGG pathways with *p* < 0.05 were considered significantly enriched.

### 4.7. Construction of Immune-Related DEmiR-DEmR Network

The DEmiRs and differentially expressed genes (DEGs) identified in a previous study [33] were used for target relationship prediction in this study using three softwares, i.e., RNAhybrid (v2.1.2) + svm_light (v6.01), Miranda (v3.3a), and TargetScan (v7.0), and the overlapping results were used for subsequent analysis. The DEmiRs and their predicted DETmRs, whose expressions were negatively correlated, were identified as DEmiRs-DETmRs pairs. A DEmiR with a degree ≥ 5 (i.e., paired with more than five DETmRs) were considered as a hub DEmiR. The hub DEmiRs and their targeted DETmRs from the immune-related KEGG pathways were used to construct the immune-related network with Cytoscape (v3.8.0) [92].

### 4.8. Identification of Interactive circRNA-miRNA and circRNA-miRNA-mRNA Regulatory Units

The target miRNAs of circRNAs were predicted based on the intersecting results of the analyses using three softwares, i.e., RNAhybrid (v2.1.2) + svm_light (v6.01), Miranda (v3.3a), and TargetScan (v7.0). The circRNAs and their paired miRNAs exhibiting negatively correlated expressions were considered as miRNA-circRNA pairs. The circRNA/miRNA/mRNA regulatory triplexes were identified based on the ceRNA theory as follows: (1) The correlation in expression between mRNA and miRNA or between circRNA and miRNA was evaluated using the Spearman Rank correlation coefficient (SCC). Pairs with SCC < −0.6 were selected as negatively co-expressed circRNA-miRNA pairs or mRNA-miRNA pairs, in which both mRNA and circRNA were miRNA target genes, and all RNAs were differentially expressed. (2) The correlation in expression between a circRNA and its target mRNA was evaluated using the Pearson correlation coefficient (PCC). Pairs with PCC > 0.8 were selected as co-expressed circRNA-mRNA pairs, in which both the mRNA and the circRNA in each pair were targeted and co-expressed negatively with a common miRNA. Only the gene pairs with a *p* value less than 0.05 were selected.

### 4.9. Experimental Validation of DEmiRs

Nine DEmiRs were selected and validated via qRT-PCR. Total RNA was isolated from spleen tissue using Trizol (Invitrogen, Carlsbad, CA, USA) and used for cDNA synthesis with a Mir-X^TM^ miRNA First-Strand Synthesis Kit (TaKaRa Bio, Mountain View, CA 94043, USA). qRT-PCR was performed with SYBR Premix Ex Taq II (TaKaRa, Dalian, China) using QuantStudio 3 Real-Time PCR Systems (Thermo Fisher Scientific, Waltham, MA, USA) in a 20 μL reaction volume containing 2 μL cDNA, 10 μL SYBR Premix Ex TaqTMII (TaKaRa, Dalian, China), 0.2 μL specific forward primer (10 μM), 0.2 μL reverse primer (10 μM), and 7.6 μL water. The reaction was performed at 95 °C for 30 s, followed by 45 cycles of 95 °C for 5 s, 60 °C for 15 s, and 72 °C for 10 s. The abundance of miRNAs was normalized relative to that of 5S rRNA with 2−ΔΔCt comparative Ct method, as reported previously [93]. The primers used for PCR analysis are list in Table 5. Correlation analysis of the results of qRT-PCR and sRNA-seq was performed using function formula CORREL in Excel.

### 4.10. Data Availability

The raw sequencing reads from this article have been deposited to NCBI Sequence Read Archive (SRA) under the accession number of PRJNA691154 (miRNA data, https://www.ncbi.nlm.nih.gov/bioproject/PRJNA691154 (accessed on 11 January 2021)) and PRJNA684425 (circRNA data, https://www.ncbi.nlm.nih.gov/bioproject/PRJNA684425 (accessed on 12 December 2020)).

## 5. Conclusions

In this study, we provide a comprehensive and systematic picture of integrated RNA responses to megalocytivirus infection in a time-dependent manner. We identified a large amount of megalocytivirus-induced non-coding RNAs and their interactive targets, which, together with coding RNAs, form complicated regulatory networks involving various miRNA-mRNA, circRNA-miRNA, and circRNA-miRNA-mRNA regulatory units. Our results indicate a profound involvement of non-coding RNAs in megalocytivirus infection and host immune response, which add new insights into the regulation of antiviral immunity in teleost fish.

## Figures and Tables

**Table 1:** Summary of miRNA sequencing of the 18 samples. V2d, V6d, and V8d represent megalocytivirus-infected fish at 2, 6, or 8 days post infection (dpi), respectively. C2d, C6d, and C8d represent control fish at 2, 6, or 8 dpi, respectively. For both virus-infected and control fish, at each time point, three fish were used (indicated by the number of 1, 2, or 3, e.g., V2d-1, V2d-2, and V2d-3).

Sample Name	Clean Tags (%)	Known miRNA	Novel miRNA
V2d-1	11,837,245 (96.83%)	365	259
V2d-2	11,280,092 (96.22%)	374	239
V2d-3	9,924,270 (96.10%)	382	247
V6d-1	17,710,805 (96.20%)	518	296
V6d-2	10,365,825 (96.37%)	425	239
V6d-3	11,893,288 (96.59%)	451	272
V8d-1	12,212,869 (96.67%)	489	286
V8d-2	13,140,335 (95.92%)	365	288
V8d-3	13,010,734 (95.98%)	476	293
C2d-1	12,573,839 (96.38%)	432	248
C2d-2	8,926,038 (94.98%)	364	233
C2d-3	8,576,422 (94.70%)	368	225
C6d-1	11,653,795 (96.21%)	413	235
C6d-2	14,097,705 (97.33%)	367	260
C6d-3	12,708,286 (95.14%)	396	261
C8d-1	10,633,416 (95.26%)	351	260
C8d-2	11,449,436 (95.92%)	357	218
C8d-3	11,412,190 (94.55%)	394	228

**Table 2:** The number of up- and downregulated differential expressed miRNAs, at 2, 6, or 8 days post infection (dpi).

Time Point	Up	Down	Total
2 dpi	7	26	33
6 dpi	69	8	77
8 dpi	83	10	93

**Table 3:** The 12 DEmiRs that formed the interactive network. For each DEmiR, the fold difference in expression between virus-infected and uninfected fish at 2, 6, and 8 dpi are indicated. Degree represents the number of differentially expressed target mRNAs (DETmRs) interacting with the DEmiRs in the network.

Hub DEmiR	Fold Change (Log2)			Degree	DETmR
	2 dpi	6 dpi	8 dpi		
novel-m0263-3p		−5.95	−4.16	11	CDK1, CSF1R2, DNASE2, INHBB, IQSEC1, RAB35, RILPL1, PAI1, SCOTIN, SHISA5, TRIM25
novel-m0233-3p	−6.21		−6.09	10	CDK1, CLTC, CYC, DNASE2, GBF1, LIMP2, PML, BID, TRIM25, HSP90α.1
miR-322-x		5.66	7.14	9	ADAR, DDX3X, TNFSF14, TPOR, PIP5K1α, TUBB, TLR8, TNFRSF10, VAMP3
miR-144-y			−2.20	8	ATPeVS1, DHX58, GIT2, IQSEC1, MRC1, PTPN11, SOCS1, VPS4B
miR-409-y			6.39	7	ABCA2, DDX3X, MET, PDGFB, STAT1, TUBB, TLR8
miR-11987-x	2.50		2.64	7	ABCA2, CCL19, CYC, PDGFB, PDGFD, GCSF, LIF
miR-495-y			5.78	6	ABCA2, CAB39L, MET, PDGFB, STAT1, TNFRSF10
novel-m0108-3p			3.23	6	ABCA2, CAB39L, DDX3X, MET, TPOR, PDGFB
miR-29-x		2.15	2.12	5	ABCA2, ADAR, AP1G1, EHD2, MET
miR-194-x			4.51	5	ABCA2, DDX3X, IST1, TNFRSF10, VAMP3
miR-727-y			−1.89	5	GBF1, GIT2, IQSEC1, IRF7, TRIM25
novel-m0007-5p			5.43	5	ABCA2, CAB39L, TPOR, PDGFB, TUBB

CDK1, cyclin-dependent kinase 1; CSF1R2, macrophage colony-stimulating factor 1 receptor 2; DNASE2, deoxyribonuclease-2-alpha; INHBB, inhibin beta B chain; IQSEC1, IQ motif and SEC7 domain-containing protein 1; RAB35, Ras-related protein Rab-35; RILPL1, RILP-like protein 1; PAI1, plasminogen activator inhibitor 1; SCOTIN, protein SCOTIN; SHISA5, protein shisa-5; TRIM25, E3 ubiquitin/ISG15 ligase TRIM25; ADAR, adenosine deaminase, RNA specific; DDX3X, ATP-dependent RNA helicase DDX3X; TNFSF14, tumor necrosis factor ligand superfamily member 14; PIP5K1α, phosphatidylinositol 4-phosphate 5-kinase type-1 alpha; TUBB, tubulin beta chain; TLR8, toll-like receptor 8; TNFRSF10, tumor necrosis factor receptor superfamily member 10B; VAMP3, vesicle-associated membrane protein 3; CLTC, clathrin heavy chain 1; CYC, cytochrome c; GBF1, Golgi-specific brefeldin A-resistance guanine nucleotide exchange factor 1; LIMP2, lysosome membrane protein 2; PML, probable transcription factor PML; BID, BH3 interacting domain death agonist; HSP90α.1, heat shock protein HSP 90-alpha; ATPeVS1, V-type proton ATPase subunit S1; DHX58, probable ATP-dependent RNA helicase DHX58; GIT2, ARF GTPase-activating protein GIT2; MRC1, macrophage mannose receptor 1; PTPN11, tyrosine-protein phosphatase non-receptor type 11; SOCS1, suppressor of cytokine signaling 1; VPS4B, vacuolar protein sorting-associated protein 4B; ABCA2, ATP-binding cassette sub-family A member 2; MET, MET proto-oncogene, receptor tyrosine kinase; PDGFB, platelet-derived growth factor subunit B; STAT1, signal transducer and activator of transcription 1; CCL19, C-C motif chemokine 19; PDGFD, platelet-derived growth factor D; GCSF, granulocyte colony-stimulating factor; LIF, leukemia inhibitory factor; CAB39L, calcium-binding protein 39; TPOR, thrombopoietin receptor; AP1G1, AP-1 complex subunit gamma-1; EHD2, EH domain-containing protein 2; IST1, vacuolar protein sorting-associated protein IST1; IRF7, interferon regulatory factor 7.

**Table 4:** The number of up- and downregulated DEcircRs, at 2, 6, and 8 days post infection (dpi).

Time Point	Up	Down	Total
2 dpi	19	25	44
6 dpi	46	31	77
8 dpi	41	33	74

**Table 5:** List of primers used for qRT-PCR in this study.

MicroRNA	Forward Primer Sequence (5′-3′)	Reverse Primer Sequence (5′-3′)
novel-m0197-3p	GACCACCCCCGAGCTTCTACGA	GTATCAACGCAGAGTACTTT
novel-m0005-5p	TACCACCCCCGAGCTTCTGCGA	GTATCAACGCAGAGTACTTT
miR-731-x	AATGACACGTTTTCTCCCGGATT	GTATCAACGCAGAGTACTTT
miR-72-x	AGGCAAGATGTTGGCATAGCT	GTATCAACGCAGAGTACTTT
miR-7133-y	TAGTTTGATACACAGCACAATG	GTATCAACGCAGAGTACTTT
miR-7132-x	GACTTGGTCAAAGCTCCTCAGC	GTATCAACGCAGAGTACTTT
miR-31-x	AGGCAAGATGTTGGCATAGCT	GTATCAACGCAGAGTACTTT
miR-155-x	TTAATGCTAATCGTGATAGGGGT	GTATCAACGCAGAGTACTTT
miR-147-y	GTGTGCGGAAAAGCTTCTGCTC	GTATCAACGCAGAGTACTTT
5s	CCATACCACCCTGAACAC	CGGTCTCCCATCCAAGTA

## Data Availability

The data presented in this study are available online (described in Section 4.10).
